# TCF7L1 Regulates *LGR5* Expression in Colorectal Cancer Cells

**DOI:** 10.3390/genes14020481

**Published:** 2023-02-14

**Authors:** Carli M. King, Olivia M. Marx, Wei Ding, Walter A. Koltun, Gregory S. Yochum

**Affiliations:** 1 Department of Biochemistry & Molecular Biology, College of Medicine, The Pennsylvania State University, Hershey, PA 17036, USA; 2 Department of Surgery, Division of Colon & Rectal Surgery, Milton S. Hershey Medical Center, The Pennsylvania State University, Hershey, PA 17036, USA

**Keywords:** colorectal cancer, LGR5, spheroids, T-cell factors, TCF7L1, WNT, Wnt-responsive DNA elements

## Abstract

Mutations in components of the Wnt/β-catenin signaling pathway drive colorectal cancer (CRC), in part, by deregulating expression of genes controlled by the T-cell factor (TCF) family of transcription factors. TCFs contain a conserved DNA binding domain that mediates association with TCF binding elements (TBEs) within Wnt-responsive DNA elements (WREs). Intestinal stem cell marker, leucine-rich-repeat containing G-protein-coupled receptor 5 (LGR5), is a Wnt target gene that has been implicated in CRC stem cell plasticity. However, the WREs at the *LGR5* gene locus and how TCF factors directly regulate *LGR5* gene expression in CRC have not been fully defined. Here, we report that TCF family member, TCF7L1, plays a significant role in regulating *LGR5* expression in CRC cells. We demonstrate that TCF7L1 binds to a novel promoter-proximal WRE through association with a consensus TBE at the *LGR5* locus to repress *LGR5* expression. Using CRISPR activation and interference (CRISPRa/i) technologies to direct epigenetic modulation, we demonstrate that this WRE is a critical regulator of *LGR5* expression and spheroid formation capacity of CRC cells. Furthermore, we found that restoring *LGR5* expression rescues the TCF7L1-mediated reduction in spheroid formation efficiency. These results demonstrate a role for TCF7L1 in repressing *LGR5* gene expression to govern the spheroid formation potential of CRC cells.

## 1. Introduction

Colorectal cancer (CRC) is the third leading cause of cancer-related deaths in the United States [1]. Mutations in key components of the Wnt/β-catenin signaling pathway are found in most sporadic CRCs, with 80–90% containing mutations in adenomatous polyposis coli (*APC*) or *CTNNB1* encoding β-catenin [2,3]. The canonical Wnt/β-catenin signaling pathway regulates stem cell self-renewal and cellular proliferation within intestinal crypts to accommodate for rapid epithelial cell turnover in the gastrointestinal tract [4,5]. However, mutations in *APC* and *CTNNB1* constitutively activate the Wnt/β-catenin signaling pathway to drive CRC tumorigenesis, in part, by promoting nuclear β-catenin accumulation and deregulating Wnt target gene expression [3,6].

As a transcriptional co-activator, β-catenin binds the T-cell factor (TCF) family of transcription factors in the nucleus to regulate target gene expression [7]. To facilitate the transcriptional response, TCF family members contain a conserved high mobility group (HMG) DNA binding domain that mediates association with consensus TCF binding elements (TBE; 5′-SCTTTGATS-3′) within Wnt-responsive DNA elements (WREs) [7]. However, upon binding to WREs, the four TCF family members, TCF7, LEF1, TCF7L1, and TCF7L2, differentially regulate Wnt/β-catenin target gene expression. TCF7L2 activates or represses gene expression, while TCF7 and LEF1 are transcriptional activators and TCF7L1 is a transcriptional repressor [7,8]. While work has focused on defining the roles of TCF7L2, TCF7, and LEF1 in CRC, the role of TCF7L1 remains poorly understood. TCF7L1 has been shown to play an oncogenic role in CRC by repressing expression of known CRC tumor suppressor ephrin type-B receptor 3 (*EPHB3*) and the Wnt antagonist Dickkopf4 (*DKK4*) [9,10]. Our group has also shown that TCF7L1 and TCF7L2/β-catenin complexes dynamically regulate *MYC* expression through defined WREs during distinct stages of the cell cycle in CRC cells [11]. Overall, few target genes directly regulated by TCF7L1 have been defined, and further identification of direct novel TCF7L1 targets is crucial to understanding its oncogenic role in CRC.

Canonical Wnt/β-catenin signaling is fundamental for regulating stem cell self-renewal and cellular proliferation in intestinal crypts and the Wnt target gene, leucine-rich-repeat containing G-protein-coupled receptor 5 (*LGR5*), has been established as the bona fide cell surface marker of intestinal stem cells [12,13]. Furthermore, LGR5 is recognized as a marker of CRC stem cells and has been implicated in CRC pathogenesis as LGR5^+^ cells are accepted as the cells-of-origin in CRC [14,15]. Moreover, the intrinsic plasticity of LGR5^−^ enterocytes to revert to LGR5^+^ cells and then re-establish the cellular hierarchy upon loss or ablation of LGR5 further implicates LGR5 in both tumor initiation and metastatic outgrowth [16,17,18,19,20]. While many studies have illustrated a crucial role LGR5 in CRC tumorigenesis, there have been relatively few that address regulation of *LGR5* gene expression in CRC beyond its identification as a Wnt target gene [21,22]. Whether TCF7L1 directly regulates *LGR5* expression in CRC is unknown.

In this study, we aimed to gain a better understanding of potential mechanisms regulating CRC stem cell plasticity. We hypothesized that TCF7L1 functions as a repressor of *LGR5* expression, and, as a result, modulates spheroid formation efficiency in CRC cells. We report that TCF7L1 directly repressed *LGR5* expression by binding to a consensus TBE within a novel WRE located at its proximal promoter. Targeted epigenetic modulation at the WRE impacted endogenous *LGR5* expression and spheroid formation efficiency in HCT116 and HT-29 cells, indicating the importance of this WRE in regulating *LGR5* expression and CRC cell stemness. Furthermore, restoration of *LGR5* expression rescued TCF7L1-mediated reduction in spheroid formation efficiency. Together, these findings demonstrate a mechanism for regulation of *LGR5* gene expression by direct binding of TCF7L1 to a novel promoter-proximal WRE.

## 2. Materials and Methods

### 2.1. Analysis of the Cancer Genome Atlas (TCGA) Datasets

Colorectal adenocarcinoma (COAD) expression data were downloaded from the TCGA database. Data were normalized using R with the variance stabilizing transformation (vst) command from the DESeq2 package. *TCF7L1* and *LGR5* transcript levels from primary tumors (*n* = 952) and normal colonic tissues (*n* = 82) were analyzed.

### 2.2. Transcript Analysis in Patient-Matched Samples

Additional patient samples evaluated in this study were obtained from the Carlino Family Inflammatory Bowel and Colorectal Disease (IBCRD) biobank within the Department of Surgery, Division of Colon & Rectal Surgery at The Pennsylvania State University Milton S. Hershey Medical Center. Surgically resected colonic tissues and tumors were collected from consenting patients following protocols approved by The Pennsylvania State University College of Medicine Institutional Review Board (PRAMSHY098-057 and STUDY00014778). Patient-matched full-thickness and colonic tumors (*n* = 43) were homogenized in 2 mL of TRIzol reagent (Thermo-Fisher, #15596026, Waltham, MA, USA) using a micropestle, RNAs purified and cDNAs synthesized as previously described [23]. *TCF7L1* and *LGR5* transcripts were evaluated in qPCR reactions containing TaqMan probes and levels were normalized to *GAPDH*. Taqman probes were purchased from Thermo-Fisher (*GAPDH*, Hs02758991_g1; *LGR5,* Hs00173664_m1; *TCF7L1,* Hs00229841_m1).

### 2.3. Cell Culture

HCT116, HT-29, and SW480 human colorectal cancer cell lines were purchased from American Type Culture Collection (ATCC) and HEK293/FT cells were purchased from Invitrogen. Cell lines were grown at 37 °C in 5% CO_2_ in Dulbecco’s Modified Eagle’s Medium (DMEM, Corning, NY, USA) supplemented with 10% fetal bovine serum (FBS, Gemini Bio, Sacramento, CA, USA), 1% penicillin/streptomycin (Gibco, Waltham, MA, USA), and 2 mM Glutamax (Gibco). HEK293FT cells were maintained with 500 µg/mL G418 (VWR, Radnor, PA, USA).

### 2.4. Plasmids

pCMV6-entry-TCF7L1 was purchased from Origene (#RC208913, Rockville, MD, USA), pCW57.1-empty vector was purchased from Addgene (#41393, Watertown, NY, USA), and pCMV-Tag2B-empty vector was purchased from Stratagene (#211172-52, San Diego, CA, USA). The pCMV6-entry-TCF7L1 plasmid expresses FLAG-tagged *TCF7L1* cDNA and was engineered to contain shRNA-resistant silent mutations using the QuikChange Lightning Site-Directed Mutagenesis kit (Agilent, #210518, Santa Clara, CA, USA). *TCF7L1* cDNA containing the shRNA-resistant mutations was amplified by PCR using oligonucleotides that incorporated EcoRI/HindIII restriction sites and the resultant fragments were inserted into the pCMV-Tag2B backbone. To generate a doxycycline (Dox)-inducible system, *TCF7L1* cDNA was PCR-amplified from pCMV-Tag2B-TCF7L1 using oligonucleotides that incorporated NheI/AgeI sites and the resultant fragments were inserted in the pCW57.1-empty vector. This plasmid is referred to as pCW57.1-TCF7L1. Primer sequences used for PCR are listed in Table S1.

pCMV6-LGR5 was purchased from Origene (#RC212825) and the TOPflash luciferase reporter was purchased from Sigma-Aldrich (#21-170, St. Louis, MO, USA). To generate pCR4-TOPO-*LGR5* wild-type and mutant plasmids, a 286-base pair region of the *LGR5* proximal promoter was PCR-amplified from HCT116 genomic DNA using oligonucleotides that incorporated KpnI/NheI sites and the fragments were subcloned into the pCR4-TOPO vector (Thermo-Fisher, #450071, Waltham, MA, USA). The mutant construct was derived by mutating the TCF binding element from CTTTGAT to CGCTGAT using the Q5 site-directed mutagenesis kit (NEB, #E0554S, Ipswich, NA, USA). Primers used in these reactions are listed in Table S1. Following sequencing confirmation, both wild type and mutant KpnI/NheI fragments were inserted into the pGL3-promoter empty vector (Promega, #E1751, Madison, WI, USA). These plasmids are referred to as *LGR5*-En and *LGR5*-En (mut), respectively.

### 2.5. Stable Cell Lines

HEK293FT cells were transfected with 3 µg of lentiviral packaging plasmids (pLP1, pLP2, and pLP/VSVG, ViraPower, Invitrogen, #K497500, Waltham, MA, USA) and 3 µg of pCW57.1-TCF7L1 using Lipofectamine 2000 (Invitrogen, #11668019) according to manufacturer’s guidelines. Media containing virus was collected at 24- and 48-h following transfection and added to HCT116 and HT-29 cells for 8 h. To obtain stable cell lines, lentiviral infected HCT116 and HT-29 cells were selected using media containing 1 or 1.5 µg/mL puromycin, respectively, for 2 weeks. After selection, stable cell lines were maintained in 0.5 µg/mL puromycin. TCF7L1 expression was induced by treating cell lines with Dox (1 µg/mL) for 18 h.

### 2.6. Reverse Transcription and Real Time PCR (RT-qPCR)

For expression analyses in established CRC cell lines, RNA was isolated using TRIzol reagent (Invitrogen, Waltham, MA, USA) and cDNA was synthesized using Verso cDNA synthesis kit (Thermo-Fisher, #AB1453B) following manufacturer guidelines. *TCF7L1* and *LGR5* expression was assessed using previously described methods [24]. Data is presented as relative expression using the 2^−ΔΔCT^ method after normalization to *TUBB1* or *ACTB* housekeeping genes. Primer sequences used are listed in Table S1.

### 2.7. Western Blot

Protein extracts from whole cell lysates were isolated in RIPA buffer (Sigma-Aldrich, #R0278, St. Louis, MO, USA), quantified and analyzed by Western blot as previously described [25]. Blots were probed with primary antibodies against TCF7L1 (Cell Signaling Technologies, #2883S, 1:500 dilution, Danvers, MA, USA), FLAG (Sigma-Aldrich, #F1804, 1:1000 dilution) and ɑ-tubulin (Sigma-Aldrich, #T9026, 1:1000 dilution). ImageJ was used for quantification.

### 2.8. Chromatin Immunoprecipitation (ChIP)

ChIP assays were conducted using the ChIP-IT High Sensitivity Kit (Active Motif, #53040, Carlsbad, CA, USA) on control (−Dox) and TCF7L1-expressing (+Dox) HCT116 cells. The cross-linked and sheared chromatin was precipitated with 4 µg of anti-FLAG antibodies (Sigma-Aldrich, #F1804). Precipitated DNA was amplified using qPCR in triplicate with primers listed in Table S1 and data is presented as percent input.

### 2.9. Luciferase Reporter Assays

Luciferase assays were conducted as previously described [26]. Cells were seeded in quadruplicate in a 24-well plate (5 × 10^4^ cells per well) and transfected with Lipofectamine 2000. Each reaction contained 100 ng of the luciferase reporter plasmid and 2 ng pLRL-SV40 *Renilla*, serving as a transfection control. Total concentration of DNA was adjusted to 500 ng per reaction using pBluescript II SK (-). Where indicated, 50 ng of pcDNA3.1-β-catenin S45F and 50 ng of pME18-Lef1 were added to the transfection reaction. Transfection media were replaced after 6 h. For Dox-induced TCF7L1 or active Wnt signaling experiments, transfection media were replaced with Dox-treated media and/or Wnt3a-conditioned media (L-Wnt3a, ATCC, CRL-2647, Gathersburg, MD, USA), respectively. After 24 h, cells were lysed in passive lysis buffer (Biotium, #99821, Fremont, CA, USA) and luciferase levels were measured using the dual luciferase single tube assay kit (Biotium, #30081) on a Glomax 20/20 single chamber luminometer (Promega, Madison, WI, USA).

### 2.10. DNA Binding Assay

The DNA binding assay was conducted as previously described [23,24]. A pair of oligonucleotides were designed with 10 nucleotides flanking both sides of the consensus TCF binding element (5′-CTTTGAT-3′) within the *LGR5* proximal promoter. The oligonucleotides were synthesized with the 5′ nucleotide biotinylated. A second pair of biotinylated oligonucleotides was synthesized using a mutant TCF binding element (5′-CGCTGAT-3′). Oligonucleotide sequences are listed in Table S1. Oligonucleotide pairs were annealed as described previously [24]. The annealed oligonucleotides were incubated with streptavidin coated magnetic beads (Promega, Z5481). Protein lysates from Dox-induced TCF7L1 HCT116 cells were isolated in RIPA buffer and 200 µg of lysate was incubated with 100 µg salmon sperm to reduce non-specific binding. An aliquot of lysate was reserved for input. Remaining lysate was incubated with the oligonucleotide probes conjugated to magnetic beads for 2 h at 4 °C. Protein/oligonucleotide complexes were captured using a magnetic stand. Proteins were eluted in Laemmli loading buffer and analyzed by Western blot using anti-FLAG antibodies (Sigma-Aldrich, #F1804, 1:1000 dilution).

### 2.11. Spheroid Formation Assays

Control (− Dox) or TCF7L1-expressing (+ Dox) HCT116 and HT-29 cells were plated on 24-well, ultra-low attachment plates (Corning, #3473, Corning, NY, USA) at a density of 500 cells per well in six technical replicates. Cells were grown in DMEM/F12 (Gibco, Waltham, MA, USA) supplemented with 2% B27 (Life Technologies, Carlsbad, CA, USA), 1 U/mL penicillin/streptomycin, 10 ng/mL human recombinant basic fibroblast (bFGF, Stem Cell, Vancouver, Canada) and 10 ng/mL epidermal growth factor (hrEGF, Stem Cell). Fresh media, supplemented with Dox (1 µg/mL) for TCF7L1-expressing cell lines, were added every 3–4 days and spheroids larger than 50 µm in diameter were quantified at 4 days (HCT116) or 8 days (HT-29) after seeding. Data is presented as spheroid formation efficiency ((total number of spheroids per well/total number of cells seeded) × 100%). Representative spheroids were imaged using an inverted, brightfield Revolve microscope (Echo, San Diego, CA, USA). For rescue experiments, 2 µg of pCMV6-LGR5 was transfected in Dox-induced TCF7L1 HCT116 or HT-29 cells using Lipofectamine LTX (Thermo-Fisher, #15338100, Waltham, MA, USA) according to manufacturer’s guidelines prior to seeding spheroid formation assays.

### 2.12. CRISPR Activation/Interference (CRISPRa/i)

The phU6-sgRNA (#53188), pLV hU6-sgRNA hUbC-dCas9-KRAB-T2a-Puro (#71236; hereafter, dCas9-KRAB), and pcDNA-dCas9-p300-Core (#61357; hereafter, dCas9-p300^CORE^) were obtained from Addgene (Watertown, NY, USA). The CRISPR guide RNA design tool, CRISPOR [27], was used to identify four guide sequences along a 300-base pair region of the *LGR5* proximal promoter containing a TCF binding element. To facilitate subcloning of the guides into the phU6-sgRNA plasmid vector, oligonucleotides encoding each guide and its corresponding reverse compliment were designed to incorporate BbsI restriction sites. Sequences used for guide RNAs can be found in Table S1. Each guide and its reverse compliment (10 µm) were annealed in 1X T4 ligase buffer and 500 U/mL T4 polynucleotide kinase by incubating the reaction for 37 °C for 30 min, followed by heating to 95 °C for 5 min and cooling to 5 °C/minute to 25 °C. Phosphorylated and annealed guide pairs were digested with BbsI and incubated with BbsI digested phU6-sgRNA plasmid, 1X T4 ligase buffer, and 1 U/µL T4 ligase for 10 min at room temperature to generate plasmids expressing four independent guide RNAs.

In the CRISPRa/i assays, 2 µg of dCas9-KRAB or dCas9-p300^CORE^ and 500 ng of each guide RNA expressing plasmid were transfected in HCT116 or HT-29 cells using Lipofectamine LTX (Thermo-Fisher, #15338100) according to the manufacturer’s protocols. After 24 h, cells were seeded for spheroid formation assays and *LGR5* expression was assessed by RT-qPCR. For rescue experiments utilizing the CRISPR activation system, TCF7L1-expressing HCT116 or HT-29 cells were transfected with 2 µg dCas9-p300^CORE^ and 500 ng of each guide RNA expressing plasmid as described above prior to seeding spheroid formation assays.

### 2.13. Statistics

The data are presented as the mean ± the standard error of the mean (SEM). Each experiment was repeated at least three times. Statistical significance was calculated using a Student’s t-test or one-way ANOVA followed by a Tukey’s or Dunnett’s test for multiple comparisons. A *p*-value < 0.05 was considered statistically significant and indicated as follows: *, *p* < 0.05; **, *p* < 0.01; ***, *p* < 0.001.

## 3. Results

### 3.1. TCF7L1 Expression Is Downregulated and LGR5 Expression Is Upregulated in Patient Tumor Samples

Using a dominant negative TCF4 (also known as TCF7L2) screen in CRC cells, van de Wetering et al. identified *LGR5* as a Wnt/β-catenin target gene [12]. Our group and others implicated direct regulation of *LGR5* expression by this pathway by localizing β-catenin binding to the *LGR5* proximal promoter and transcriptional start site using chromatin immunoprecipitation (ChIP) assays [21,22,26,28,29]. However, because mutations render Wnt/β-catenin signaling constitutively active in CRCs, the factors or mechanisms involved in repressing *LGR5* expression have remained elusive. We therefore hypothesized that the TCF family member, TCF7L1, might directly repress *LGR5* expression through a promoter-proximal WRE.

**Figure 1:**
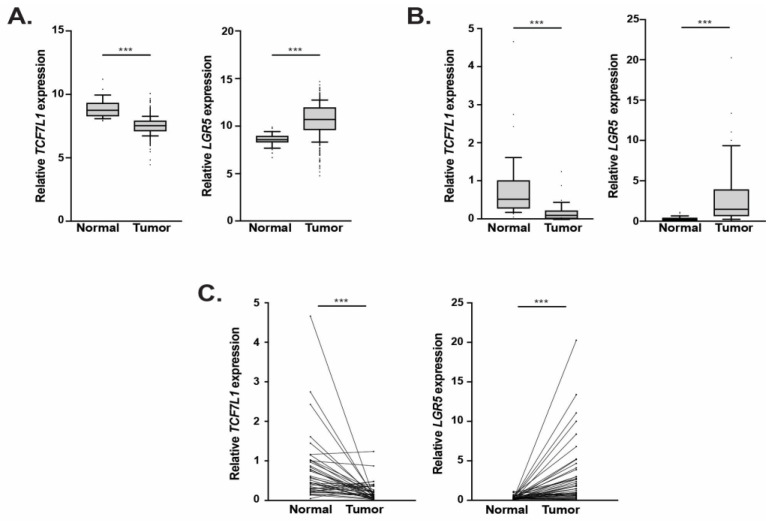
*TCF7L1* expression is downregulated and *LGR5* expression is upregulated in patient tumor samples. (**A**) Relative *TCF7L1* and *LGR5* transcript levels in normal colons and tumor patient samples within TCGA COAD database. (**B**) Relative *TCF7L1* and *LGR5* transcript levels in normal and tumor patient-matched samples obtained from the Carlino Family Inflammatory Bowel and Colorectal Disease biobank at the Pennsylvania State University College of Medicine. Values are normalized to *GAPDH.* (**C**) Pair-wise analysis of *TCF7L1* and *LGR5* transcript levels in samples described in (**B**). In (**A**,**B**), whiskers demarcate the 10–90 percentile range (*** *p* < 0.001).

To begin to test this hypothesis, we first determined whether there was an association between *TCF7L1* and *LGR5* expression in tumor and normal colonic tissue samples available in the colorectal adenocarcinoma (COAD) data set within The Cancer Genome Atlas (TCGA) database. Compared to normal tissues, *TCF7L1* expression was decreased and *LGR5* expression was increased in tumors (Figure 1A). However, the COAD dataset is limited by fewer normal colonic samples (*n* = 82) than tumor samples (*n* = 952) and the lack of patient-matched samples. To address these limitations in an independent cohort, we evaluated *TCF7L1* and *LGR5* expression in patient-matched samples (*n* = 43) available within our Inflammatory Bowel and Colorectal Disease (IBCRD) biobank. As was the case with the TCGA analysis, *TCF7L1* expression was decreased and *LGR5* expression was increased in tumors relative to control tissues (Figure 1B). Importantly, this correlation was seen when *TCF7L1* and *LGR5* transcripts were compared within each independent patient sample set (Figure 1C). Together, these results identify an inverse correlation between *TCF7L1* and *LGR5* expression in tumors and suggest that TCF7L1 could play a direct or indirect role in repressing *LGR5* expression in CRC.

### 3.2. TCF7L1 Represses LGR5 Expression and Directly Binds the LGR5 Promoter Region

**Figure 2:**
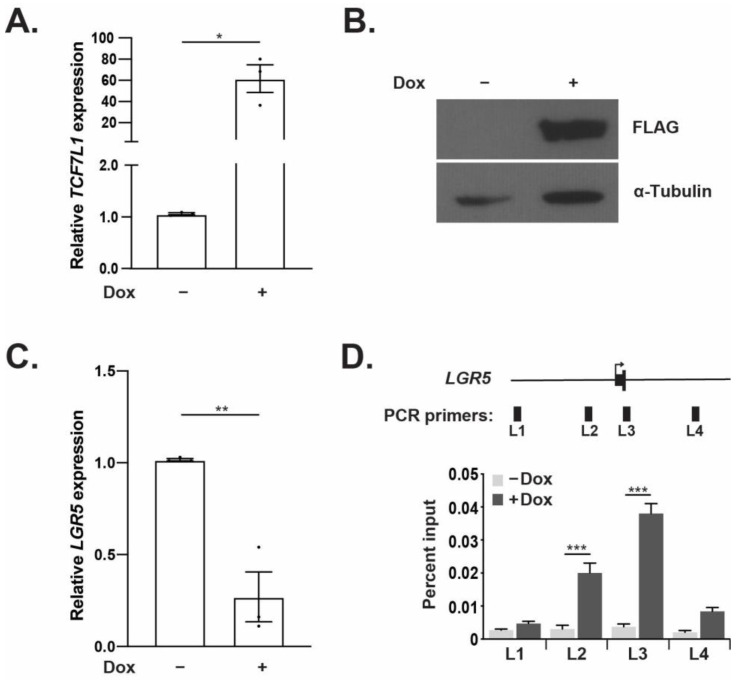
TCF7L1 represses *LGR5* expression and directly binds to the *LGR5* promoter region. (**A**) RT-qPCR and (**B**) Western blot analyses of TCF7L1 expression in untreated (−) or Dox-treated (+) FLAG-TCF7L1 HCT116 cells. (**C**) RT-qPCR analysis of *LGR5* transcripts in FLAG-TCF7L1 HCT116 cells ± Dox. (**D**) Diagram of the *LGR5* gene with the primer positions used for qPCR indicated by black rectangles. ChIP-qPCR analysis of anti-FLAG immunoprecipitated and purified DNA in FLAG-TCF7L1 cells ± Dox. Relative expression values are normalized to *TUBB1.* ChIP data are presented as percent of input. Error bars represent SEM (* *p* < 0.05, ** *p* < 0.01, *** *p* < 0.001).

To test whether TCF7L1 regulates *LGR5* expression in CRC, we engineered HCT116 and HT-29 established CRC cell lines to express a FLAG epitope-tagged *TCF7L1* cDNA in a doxycycline (Dox)-inducible manner. HCT116 cells treated with Dox showed a 60-fold increase in *TCF7L1* expression at the transcript level when compared to untreated controls (Figure 2A). Western blot analysis found that Dox treatment also increased TCF7L1 protein levels (Figure 2B). In addition, stable FLAG-TCF7L1 HT-29 cells also displayed a Dox-induced increase in TCF7L1 transcripts and proteins (Supplemental Figure S1A,B). Endogenous *LGR5* expression was significantly reduced after Dox treatment relative to untreated controls in both HCT116 (Figure 2C) and HT-29 (Supplemental Figure S1C) cell lines. Together, these findings indicate that TCF7L1 directly or indirectly represses *LGR5* expression.

Using previously published ChIP-based screens identifying β-catenin-bound genomic regions in HCT116 cells, we identified a region of DNA bound by β-catenin near the transcriptional start site (TSS) of the *LGR5* gene [26,28,29]. To determine whether TCF7L1 bound the *LGR5* gene locus, we conducted ChIP-qPCR assays in the stable FLAG-TCF7L1 HCT116 cell line. We designed primer sets that tiled the *LGR5* promoter, TSS, first exon and first intron of *LGR5* to measure potential TCF7L1-occupancy at these sites in anti-FLAG immunoprecipitated and purified DNA (designated L1-L4, Figure 1D). We detected little TCF7L1 binding to regions L1-L4 in the absence of Dox (control), whereas we detected strong TCF7L1 binding regions L2 and L3 with moderate binding at L4 in Dox-treated cells (Figure 1D). Upon analysis of DNA sequences within primer sets L2 and L3, we identified a consensus TCF binding element (TBE; 5′-SCTTTGATS-3′) within the L2 fragment. Because binding of β-catenin/TCF7L2 at the TSS has been described [26,28,29], in subsequent experiments we focused our attention to the upstream TCF7L1 binding site within the *LGR5* promoter (denoted as L2) as a second potential site for TCF function.

### 3.3. TCF7L1 Occupancy Demarcates a WRE at the LGR5 Locus That Requires a Single TBE for Full Activity

**Figure 3:**
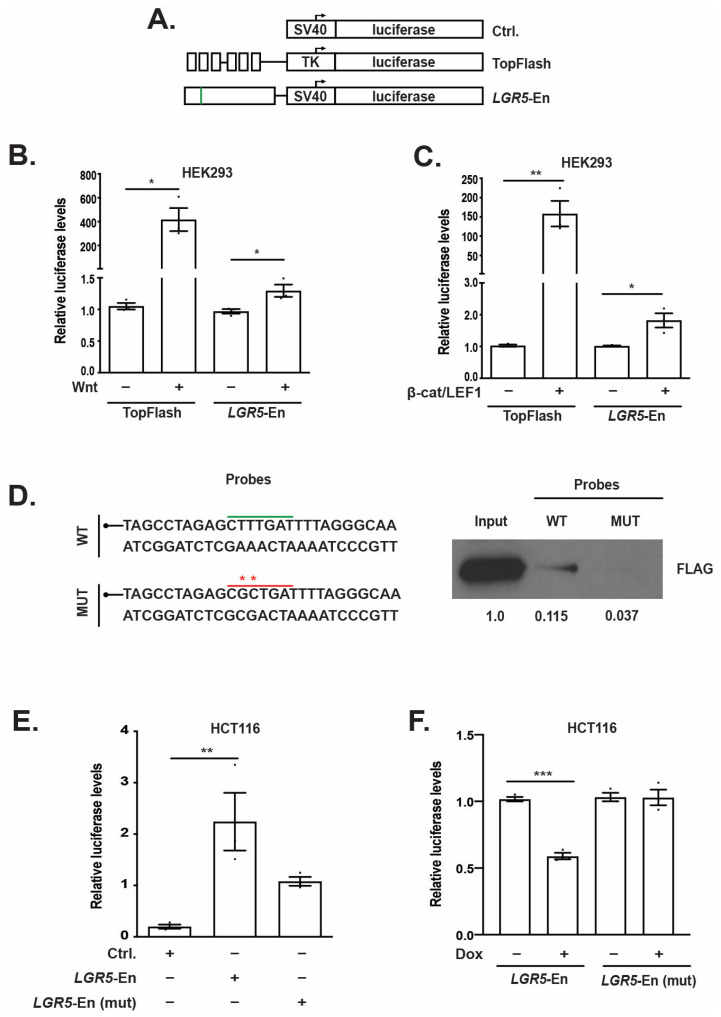
TCF7L1 occupancy demarcates a WRE at the *LGR5* locus that requires a TBE for full activity. (**A**) Diagram of luciferase reporter plasmids. (**B**) Luciferase levels expressed in HEK293 cells transfected with a reporter plasmid harboring a region of the *LGR5* proximal promoter (*LGR5*-En) or TOPflash. Where indicated, cells were treated with Wnt3a-conditioned media. (**C**) As in (**B**), except HEK293 cells were or co-transfected with plasmids encoding β-catenin and LEF1 as indicated. **(D**) DNA sequence of binding assay probes flanking the consensus TBE (underlined in green for wild-type sequence or red for mutant sequence) with the engineered mutations indicated by asterisks. Western blot analysis of FLAG-tagged TCF7L1 from FLAG-TCF7L1 HCT116 protein lysates that were incubated with the biotinylated DNA probes indicated. (**E**) Luciferase levels expressed in HCT116 cells transfected with control (Ctrl.), *LGR5*-En, or *LGR5*-En (mut). (**F**) Luciferase levels expressed in control (− Dox) or Dox-treated FLAG-TCF7L1 HCT116 cells transfected with *LGR5*-En or *LGR5*-En (mut), as indicated. Error bars represent SEM (* *p* < 0.05, ** *p* < 0.01, *** *p* < 0.001).

Our lab and others have previously demonstrated that TCF7L1 functions as a transcriptional repressor in CRC cells [9,10,11] and that it can directly repress WRE-mediated gene expression [11]. We therefore hypothesized that the identified TCF7L1-binding region within the *LGR5* promoter region demarcated a WRE. To test this, we generated a luciferase reporter construct containing a 286-base pair segment incorporating the consensus TBE within the *LGR5* promoter region inserted upstream of the minimal SV40 promoter in the pGL3-promoter luciferase vector, termed *LGR5*-En (Figure 3A). The Wnt-responsive TOPflash reporter plasmid was used as a positive control and the pGL3-promoter vector was used as a negative control in some experiments (Figure 3A). Treatment of HEK293 cells with Wnt3A-conditioned media stimulated *LGR5*-En activity (Figure 3B). Similarly, co-transfection of plasmids expressing β-catenin and LEF1 also increased *LGR5*-enhancer driven luciferase levels (Figure 3C). In addition, *LGR5*-En displayed increased activity versus empty vector controls in the CRC cell lines HT-29 and SW480 that contain constitutively active Wnt/β-catenin signaling (Supplemental Figure S2). These results demonstrate that the DNA segment that was bound by TCF7L1 (Figure 2D) is a functional Wnt-responsive DNA enhancer element.

We next utilized a DNA binding assay to determine whether TCF7L1 could bind to the segment of the WRE containing the consensus TBE motif and whether mutations in this motif would block TCF7L1 binding. Protein lysates from Dox-treated FLAG-TCF7L1 HCT116 cells were incubated with biotinylated DNA probes and protein/DNA complexes were captured using streptavidin-conjugated magnetic beads. Eluted proteins were analyzed by Western blot, and we found that TCF7L1 bound this TBE within the WRE (Figure 3D). Mutating the consensus motif TBE strongly reduced TCF7L1 binding to this fragment of DNA (Figure 3D). We then engineered these same TBE mutations within the enhancer region of *LGR5*-En luciferase construct to generate *LGR5*-En (mut). In transfected HCT116 cells, we noted high activity of *LGR5*-En, versus empty vector control, which is likely due to binding of TCF/β-catenin complexes to the enhancer (Figure 3E). The *LGR5*-En (mut) construct expressed less luciferase compared to *LGR5*-En indicating that the wild-type TBE motif is required for full WRE activity (Figure 3E). In similar assays conducted in the Dox-inducible FLAG-TCF7L1 HCT116 cells, TCF7L1 repressed *LGR5*-En and this activity was not seen in cells transfected with *LGR5*-En (mut) (Figure 3F). In these cells, Dox treatment also reduced TOPflash luciferase levels as a control for TCF7L1 activity (Supplemental Figure S3). These data indicate that TCF7L1 represses the *LGR5* WRE through association with an embedded TBE.

### 3.4. TCF7L1 Reduces Spheroid Formation Efficiency of CRC Cell Lines

**Figure 4:**
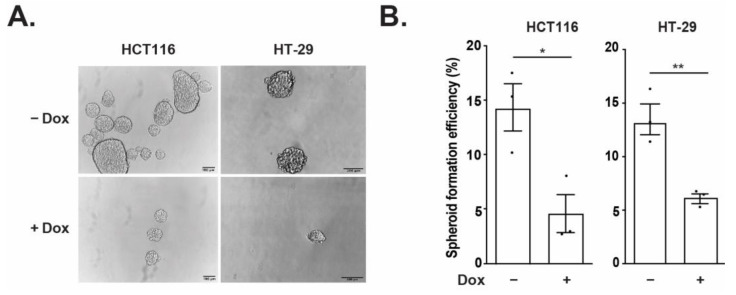
TCF7L1 reduces spheroid formation efficiency of CRC cell lines. (**A**) Representative images of spheroids formed in untreated (ctrl) and Dox-treated FLAG-TCF7L1 HCT116 and FLAG-TCF7L1 HT-29 cell lines. Scale bars are 100 µm. (**B**) Average spheroid formation efficiency of spheroids derived from FLAG-TCF7L1 HCT116 and FLAG-TCF7L1 HT29 cells ± Dox treatment. Data are represented as mean ± SEM. (* *p* < 0.05, ** *p* < 0.01).

When CRC cells are seeded at low density on ultra-low attachment plates they grow as three-dimensional spheroids that are enriched in stem cells, which impart self-renewal capacity and clonal growth capabilities [30,31]. Previous studies have found that *LGR5* expression is enriched in spheroid models and that silencing *LGR5* reduces spheroid forming potential [15,32]. To assess the functional implications of TCF7L1 in repressing *LGR5* transcription, we measured the spheroid formation efficiency of FLAG-TCF7L1 HCT116 and FLAG-TCF7L1 HT-29 cells. In comparison to untreated controls, Dox-induced TCF7L1 caused a significant reduction in spheroid formation efficiency in both cell lines (Figure 4A,B). These data suggest that TCF7L1 repression of *LGR5* may play an important role in CRC stem cell function in spheroid cultures.

### 3.5. Epigenetic Regulation of the WRE Impacts LGR5 Expression and Spheroid Formation

**Figure 5:**
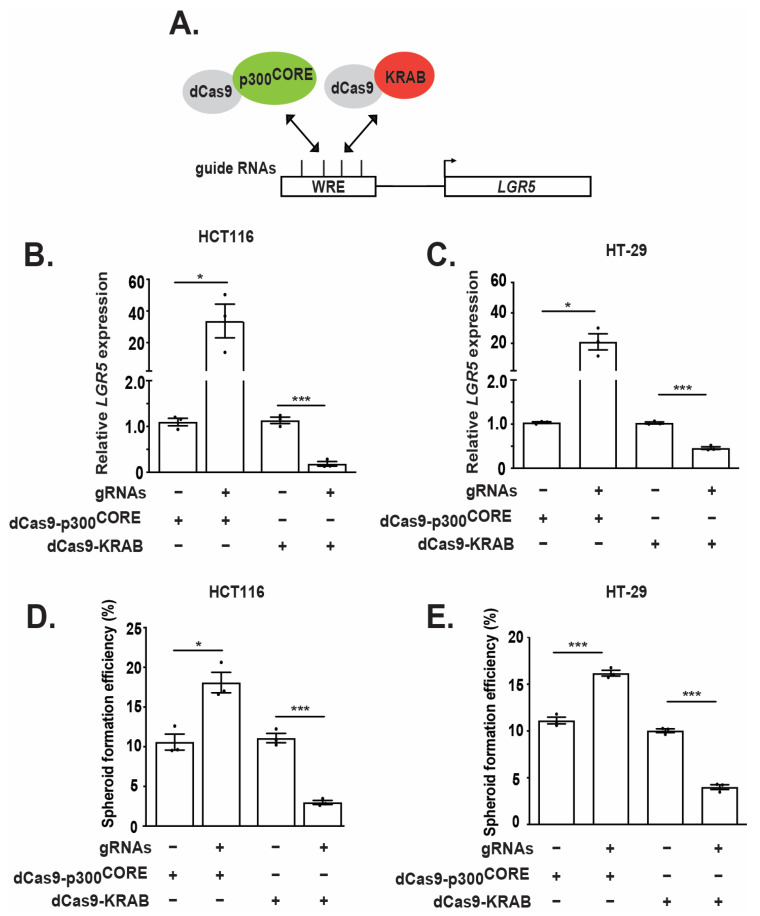
Epigenetic regulation of the WRE impacts *LGR5* expression and spheroid formation. (**A**) Diagram of CRISPR activation and interference systems. The relative positions of each guide RNA within the WRE region upstream of the *LGR5* locus is denoted with black vertical lines. (**B**,**C**) RT-qPCR analysis of *LGR5* transcripts and (**D**,**E**) spheroid formation efficiency of HCT116 and HT-29 cells. As indicated, cells were co-transfected with plasmids expressing dCas9-KRAB or dCas9-p300^CORE^ ± guide RNAs. *LGR5* expression values are normalized to *ACTB.* Data are represented as mean ± SEM (* *p* < 0.05, *** *p* < 0.001).

To determine whether the defined promoter-proximal WRE is a critical regulator of endogenous *LGR5* expression, we employed a CRISPR activation and interference (CRISPRa/i) approach to epigenetically activate or repress this DNA regulatory element in CRC cell lines [33]. Briefly, this assay utilizes guide RNAs (gRNAs) to recruit a mutant Cas9 (dCas9), lacking endonuclease activity, fused to the p300^CORE^ subunit (dCas9-p300^CORE^) or the KRAB transcriptional repressor domain (dCas9-KRAB) to the DNA region of interest (Figure 5A). For these experiments, four gRNAs tiling the 286-base pair region defined as the WRE (Figure 2 and Figure 3) were generated to direct dCas9-p300^CORE^ or dCas9-KRAB to the endogenous *LGR5* locus (Figure 5A). HCT116 cells co-transfected with plasmids expressing the four gRNAs and dCas9-p300^CORE^ or dCas9-KRAB displayed a significant increase or decrease in *LGR5* transcripts, respectively, relative to controls lacking gRNAs (Figure 5B,C). Similarly, gRNA/dCas9-KRAB complexes reduced *LGR5* transcript levels, while gRNA/dCas9-p300^CORE^ complexes increased *LGR5* transcript levels, in HT-29 cells relative to control cells (Figure 5C). Furthermore, CRISPR activation of *LGR5* expression increased, whereas inactivation decreased, the spheroid-formation efficiency of both cell lines (Figure 5D,E). Together, these data demonstrate that the WRE regulates *LGR5* expression and functionally impacts spheroid formation efficiency in CRC cells. These data are in alignment with previous studies illustrating that *LGR5* expression directly impacts spheroid formation [15,32].

### 3.6. LGR5 Rescues TCF7L1-Mediated Reduction in Spheroid Formation Efficiency

**Figure 6:**
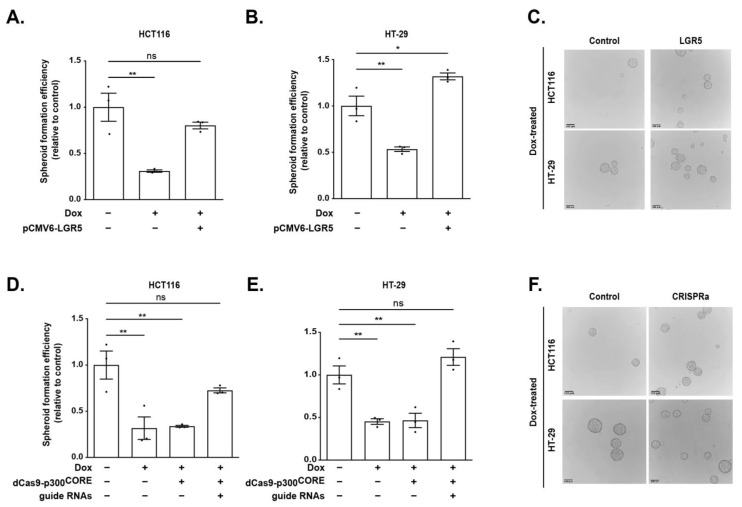
*LGR5* expression rescues TCF7L1-mediated reduction in spheroid formation efficiency. (**A**,**B**) Spheroid formation efficiency and (**C**) representative images of spheroids formed from FLAG-TCF7L1 HCT116 and HT-29 cell lines that were transfected with a plasmid expressing *LGR5* cDNA as indicated. (**D**,**E**) Spheroid formation efficiency and (**F**) representative images of spheroids formed from FLAG-TCF7L1-expressing cells that were co-transfected with plasmids expressing dCas9-p300^CORE^ ± guide RNAs as indicated. Scale bars are 100 µm. Data are normalized to spheroid formation efficiency of untreated HCT116 or HT-29, respectively, and represented as mean ± SEM (* *p* < 0.05, ** *p* < 0.01).

We next conducted rescue experiments to determine whether LGR5 was required for the decreased spheroid formation efficiency seen in TCF7L1-overexpressing CRCs. As reported earlier (Figure 4B), Dox-induced FLAG-TCF7L1 expression resulted in a low spheroid-generating phenotype (~5%) in HCT116 and HT-29 cells (Figure 6A,B). In parallel cultures, introduction of a plasmid expressing *LGR5* cDNA restored the spheroid capacity of these cells when they were cultured in the presence of FLAG-TCF7L1 (Figure 6A–C). To determine whether increasing *LGR5* expression through the WRE would likewise rescue these phenotypes, we utilized CRISPRa. Targeting the WRE through specific gRNAs and dCas9-p300^CORE^ in FLAG-TCF7L1-expressing cells significantly increased spheroid formation efficiency of both HCT116 and HT-29 cells (Figure 6D–F). Taken together, these data demonstrate that *LGR5* expression fully rescues the TCF7L1-mediated reduction in spheroid formation efficiency in both HCT116 and HT-29 cell lines. Furthermore, these data indicate that TCF7L1 likely plays a role in regulating CRC cell stemness through direct repression of *LGR5* expression and highlights the importance of the WRE controlling this function.

## 4. Discussion

Activating mutations in the Wnt/β-catenin signaling pathway leads to deregulation of Wnt target gene expression that drives CRC pathogenesis [6]. As the TCF family of transcription factors are critical mediators of Wnt target gene expression, an emphasis has been placed on elucidating the mechanism by which each member contributes to CRC progression. Reports regarding TCF members often contribute to defining the roles of TCF7L2, TCF7, or LEF1, with little focus on addressing the role of TCF7L1 in CRC. Recent studies have implicated TCF7L1 as an oncogene in CRC; however, very few target genes have been identified [9,10,11]. To further define the role of TCF7L1 as an oncogene in CRC, it is crucial to identify the targets of TCF7L1 regulation. In this report, we expanded the list of direct TCF7L1 targets to include *LGR5*.

Among the few known TCF7L1 targets are tumor suppressor genes *DKK4* and *EPHB3*, which is consistent with the role of TCF7L1 as an oncogene in CRC [9,10]. However, previous analyses of transcriptome data indicate that *TCF7L1* transcripts are downregulated in human colorectal cancers versus normal colonic control tissues [34,35]. We confirmed these findings in our analyses of TCGA data and tissue samples within our Carlino Family IBCRD biorepository (Figure 1). While this expression pattern appears contradictory to its function as an oncogene, there are at least two plausible explanations to explain this discrepancy. First, we and others have found abundant expression of TCF7L1 proteins in established human colorectal cancer cell lines indicating that despite lower levels of transcripts, functional protein is expressed [9,10,11,36]. It is therefore likely that post-translational regulation of TCF7L1 stability is more critical to support its oncogenic function in these cells than upregulation of *TCF7L1* mRNA at the transcriptional or post-transcriptional level. A comprehensive analysis of TCF7L1 proteins in patient-matched tumors and control colonic tissue by Western blot and immunohistochemical staining is needed to resolve this issue. Second, analysis of bulk RNA transcripts does not allow for the same granularity as single-cell sequencing for evaluating expression in subpopulations of cells in heterogeneous tumor samples. TCF7L1 may play a specific role in repressing *LGR5* expression in a subpopulation of cells to regulate cancer stem cell plasticity. Our findings that *TCF7L1* transcripts are inversely correlated with *LGR5* transcripts in CRC support a role for TCF7L1-mediated *LGR5* repression. Thus, despite the downregulation of *TCF7L1* transcripts in tumors, our data further support an oncogenic role for TCF7L1 in CRC.

The Wnt target gene, *LGR5,* has been recognized as the bona fide cell surface marker of intestinal stem cells and its expression has been used to isolate and identify CRC stem cells [13,15,17]. CRC stem cells contain mutations in the Wnt/β-catenin signaling pathway, most often in the *APC* tumor suppressor, that drive target gene expression through TCF/β-catenin complexes. Yet, expression of the Wnt target gene *LGR5* must be precisely tuned to facilitate plasticity at distinct stages of colorectal cancer pathogenesis. How *LGR5* expression is repressed in the context of constitutively active Wnt signaling in CRC remains largely unknown. We propose that TCF7L1 can repress *LGR5* expression despite constitutive Wnt/β-catenin signaling driven by pathway mutations in these cells.

As a Wnt target gene, β-catenin/TCF7L2 binding sites have been identified near the TSS at the *LGR5* locus [26,28]. In cooperation with β-catenin/TCF7L2 complexes, the methyltransferase Mll1 and Tribbles pseudo-kinase 3 (TRIB3) promote CRC stem cell phenotypes by increasing transcription activity at the *LGR5* locus [22,37]. However, the WREs governing regulation at the *LGR5* gene locus and how the TCF family of transcription factors directly regulate *LGR5* expression in CRC were not fully defined. In this study, we define a novel WRE at the *LGR5* proximal promoter that was subjected to regulation by TCF7L1. Here, we propose two potential mechanisms for cooperation or competition between TCF7L1 and TCF7L2/β-catenin complexes to regulate *LGR5* expression in CRC. The *LGR5* WRE may undergo temporal regulation by TCF7L1 and TCF7L2/β-catenin, similar to the mechanism previously described at the 3′ *MYC* WRE [11]. Alternatively, the WRE may be regulated by TCF7L1, in parallel to TCF7L2/β-catenin regulation at the *LGR5* TSS, for a combinatory effect to fine-tune *LGR5* expression. In addition, GATA6, DEAD box RNA helicase protein DDX1, Jade family PHD finger 3 (JADE3) have been implicated as direct activators of *LGR5* expression, while Dickkopf-2 (DKK2) indirectly activates *LGR5* expression by promoting downstream degradation of hepatocyte nuclear factor 4-α (HNF4ɑ1) in CRC [38,39,40,41,42]. Together, these findings suggest that the Wnt/β-catenin signaling pathway may intersect other signaling pathways to provide multiple mechanisms for regulating *LGR5* expression. Whether TCF7L1 cooperates with or antagonizes the activity of these other transcription factors to modulate *LGR5* expression is an important avenue of further investigation that will require analysis of LGR5^−^ and LGR5^+^ CRC stem cells.

Additional work is needed to address the mechanism by which TCF7L1 represses *LGR5* expression. Our previous study demonstrated that TCF7L1 recruits histone deacetylase 1 (HDAC1) and co-repressor C-terminal binding protein (CtBP) to the *DKK4* promoter to repress *DKK4* expression and in the absence of TCF7L1, TCF7L2/β-catenin complexes occupy the DKK4 promoter to activate *DKK4* expression [9]. Whether this, or distinct mechanisms of TCF-mediated regulation occur at the *LGR5* locus are critical areas of future research.

Our study further defines a role for TCF7L1 in cancer stem cell biology by demonstrating that TCF7L1 mediates repression of *LGR5* expression and that this contributes to reduction of spheroid formation efficiency. LGR5^+^ stem cells have been implicated in many stages of CRC tumorigenesis, from initiation as the cells-of-origin [14] to outgrowth and maintenance of metastatic lesions [17]. More recently, LGR5^−^ cancer cells have been distinguished as the majority of migrating and disseminating cells from the primary tumor to the metastatic site, highlighting an important role for LGR5^−^ cancer cells and their intrinsic capability to re-establish the LGR5^+^ cancer cell population [20]. This is further supported by evidence of both LGR5^+^ and LGR5^−^ stem cell populations in CRC tumors and the shifting of these populations based upon selective pressures, such as chemotherapies [43]. We predict that TCF7L1 levels will be highest in LGR5^−^ cells and lower in LGR5^+^ contributing to the epithelial hierarchy of CRC.

There are several strengths of our study including an evaluation of primary human tissue samples and tumors, use of multiple established CRC cell lines, and the use f rescue experiments to support the TCF7L1/LGR5 pathway as a critical regulator of CRC stemness. However, our study is limited by the lack of in vivo murine tumor models and evaluation of cancer stem cells in human tumoroid (organoid) systems. Incorporation of these models in future work will allow us to better understand how Wnt/β-catenin signaling and TCF7L1 control CRC stem cell populations to promote colorectal carcinogenesis and chemotherapeutic resistance.

The plastic nature of CRC stem cells makes them a difficult target for therapeutic intervention. We propose that the promoter-proximal WRE at the *LGR5* locus defined in this study may be a novel therapeutic target. Modulation of this WRE may provide an avenue for regulating cancer cell plasticity, which has been linked to drug resistance, tumor relapse, and metastasis [44]. Targeting of epigenetic modifiers using CRISPRa/i to the *LGR5* WRE offers a potential therapeutic strategy to improve patient care.

## Data Availability

The human colon cancer data referenced are available in public repository from The Cancer Genome Atlas colorectal adenocarcinoma data set (TCGA-COAD). All other data are available within this manuscript and its supplementary files and from the corresponding author upon request.

## Supplementary Materials

The following supporting information can be downloaded at: https://www.mdpi.com/article/10.3390/genes14020481/s1, Figure S1: TCF7L1 represses *LGR5* expression in HT-29 cells; Figure S2: The TCF7L1 binding site at the *LGR5* proximal promoter demarcates a WRE in CRC cells; Figure S3: TOPflash control expression in FLAG-TCF7L1 HCT116 cells; Table S1: List of oligonucleotide sequences.
