# Supplementary material for: TCF7L1 Regulates LGR5 Expression in Colorectal Cancer Cells
Source: Genes (Basel). 2023 Feb 14;14(2):481. doi: 10.3390/genes14020481 (PMC9956233; doi:10.3390/genes14020481)
Supplement: Supplementary file 1 [file genes-14-00481-s001.zip › genes-2145120-supplementary.pdf]

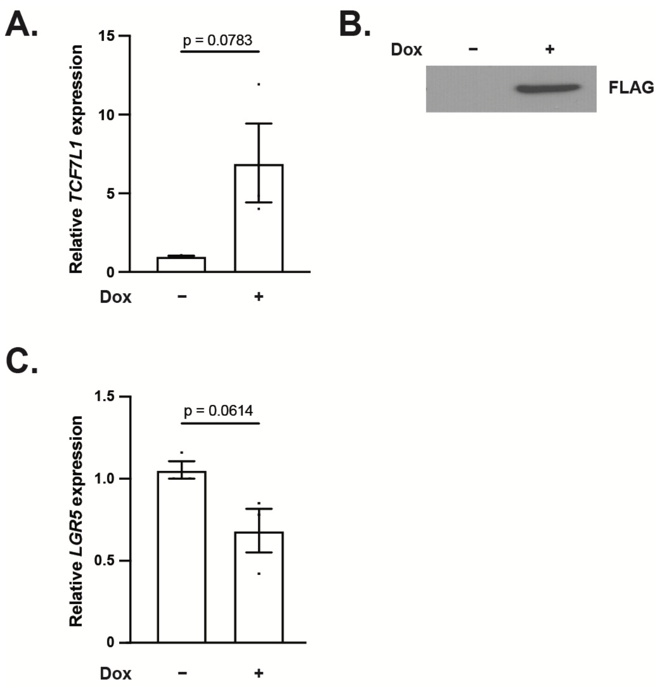

Supplemental Figure S1. TCF7L1 represses *LGR5* expression in HT-29 cells. (**A**) RT-qPCR and (**B**) western blot analyses of TCF7L1 expression FLAG-TCF7L1 HT29 cells ± Dox treatment. (**C**) RT-qPCR analysis of *LGR5* transcripts. Relative expression values are normalized to *TUBB1*. Data are represented as mean ± SEM with P-values indicated.

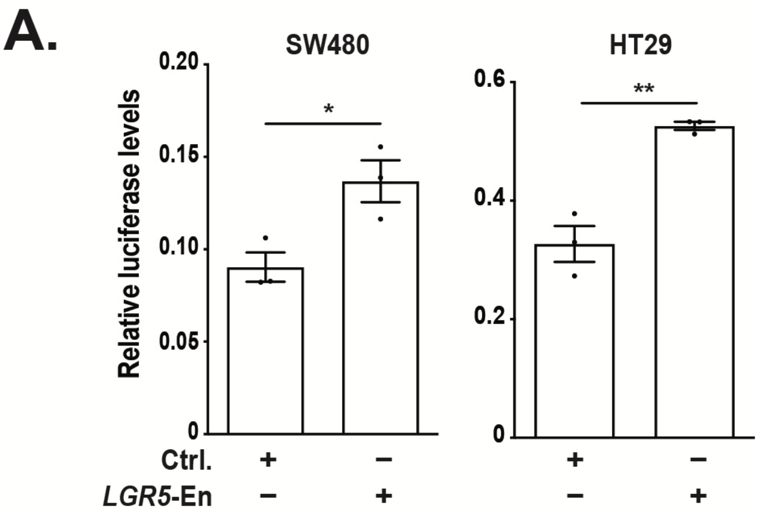

Supplemental Figure S2. The TCF7L1 binding site at the *LGR5* proximal promoter demarcates a WRE in CRC cells. (**A**) Luciferase expression levels in SW480 and HT-29 cells transfected with control or *LGR5*-En. Values are normalized to *Renilla* luciferase controls. Data are represented as mean ± SEM (*$P < 0.05$, **$P < 0.01$).

A.

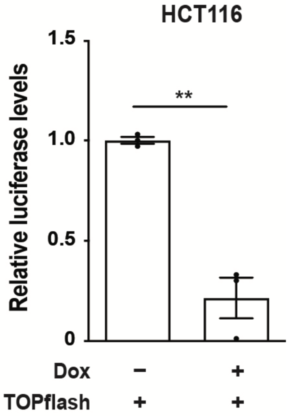

Supplemental Figure S3. TOPflash control expression in FLAG-TCF7L1 HCT116 cells. (**A**) Luciferase expression levels in control (−) and Dox-treated (+) FLAG-TCF7L1 HCT116 cells transfected with TOPflash Wnt activity reporter plasmid. Values are normalized to *Renilla* luciferase controls. Data are represented as mean ± SEM (***P* < 0.01).

Table S1: List of oligonucleotide sequences.

**TaqMan Probes**		
TCF7L1		ThermoFisher: Hs00229841_m1
LGR5		ThermoFisher: Hs00173664_m1
GAPDH		ThermoFisher: Hs02758991_g1
**Stable cell lines**		
Site-directed mutagenesis (shRNA resistance)	Forward	5' - CTG AAC GAC AGG AAC TTT ATT GGA TAA ATG TGC TGG AGA TGG TGA CCT C - 3'
	Reverse	5' - GAG GTC ACC ATC TCC AGC ACA TTT ATC CAA TAA AGT TCC TGT CGT TCA G - 3'
pCMV-Tag2b-TCF7L1 (EcoRI)	Forward	5' - CCG AAT TCC CCC AGC TCG GCG - 3'
pCMV-Tag2b-TCF7L1 (HindIII)	Reverse	5' - CCC AAG CTT TTA GTG GGC AGA CTT GGT GAC C - 3'
pCW57.1-TCF7L1 (NheI)	Forward	5' - CTA GCT AGC GCC ACC ATG GAT TAC AAG G - 3'
pCW57.1-TCF7L1 (AgeI)	Reverse	5' - TAG ACC GGT GTC GAC GGT ATC GAT AAG C - 3'
**RT-qPCR**		
TCF7L1	Forward	5' - GCC ACT CCC TCT GCA GCT TTG G - 3'
	Reverse	5' - TTT CTG GTT TGG TGG TGA GGG AGA - 3'
LGR5	Forward	5' - GCC CTG TGG CTT TCT TGT CCT TCT - 3'
	Reverse	5' - GGG ATT GAG ACA TGC AGG AAG TGG- 3'
TUBB1	Forward	5' - CAG CAC TCC AAA ACC CAC TCT GC - 3'
	Reverse	5' - ACC TGT CTC CCT CAG TCC CTG TGT - 3'
ACTB	Forward	5' - GAG CAT CCC CCA AAG TTC ACA ATG - 3'
	Reverse	5' - TGG CTT TTA GGA TGG CAA GGG ACT - 3'
**ChIP**		
L1	Forward	5' - AGG CAC GTC TTC ACA TGA GGA AGC - 3'
	Reverse	5' - TGA ATT TCC CCC TTG CTG TTC TCA - 3'
L2	Forward	5' - CTC ACC CCG CAA GAG ATA GGA AGG - 3'
	Reverse	5' - TTT GAT CCT TAC GTC TGC CGC ACT - 3'
L3	Forward	5' - GTC TCT CCG AAG CAG GTC CCT CTC - 3'
	Reverse	5' - CGC CTG GAA GAT TCA GTG CTC AAC - 3'
L4	Forward	5' - GGG GCC CAG TAG AAA TTA AGC CTT G - 3'
	Reverse	5' ACA GCT GGA ACT TGC CAT CAT CAA - 3'
**Luciferase plasmid generation**		
Region of *LGR5* promoter	Forward	5' - GGG GTA CCC CGG AGC TGT ATT TGT TTA GC - 5'
	Reverse	5’ - CTA GCT AGC TAG GAG AGA CCT AAA CAG ACG C – 3’
Q5-site directed mutagenesis	Forward	5' - GAT TTT AGG GCA AAA GCG AGC - 3'
	Reverse	5' - AGC GCT CTA GGC TAA TCG CTA AAC - 3'
**DNA binding assay**		
WT probe	Forward	5' - (biotinylated) TAG CCT AGA GCT TTG ATT TTA GGG CAA - 3'
	Reverse	5' - TTG CCC TAA AAT CAA AGC TCT AGG CTA - 3'
MUT probe	Forward	5 - (biotinylated)TAG CCT AGA GCG CTG ATT TTA GGG CAA - 3'
	Reverse	5' - TTG CCC TAA AAT CAG CGC TCT AGG CTA - 3'
**CRISPRa/i**		
gRNA set 1	Forward	5' - CAC CGC TTA CGT CTG CCG CAC TGT C 3'
	Reverse	5' - AAA CGA CAG TGC GGC AGA CGT AAG C - 3'
gRNA set 2	Forward	5' - CAC CGC GTC CCC GGC GAA TGA TAG G - 3'
	Reverse	5' - AAA CCC GAT CAT TCG CCG GGG ACG C - 3'
gRNA set 3	Forward	5' - CAC CGT TAC GTT ATC AGG GTA AGG - 3'
	Reverse	5' - AAA CCC TTA CCC TGA TAA CGT AAC - 3'
gRNA set 4	Forward	5' - CAC CGA TTA TTT GAA GCG GGC TCG G - 3'
	Reverse	5' - AAA CCC GAG CCC GCT TCA AAT AAT C - 3'
